# Factors of land abandonment in mountainous Mediterranean areas: the case of Montenegrin settlements

**DOI:** 10.1186/s40064-016-2079-7

**Published:** 2016-04-19

**Authors:** Annelies Kerckhof, Velibor Spalevic, Veerle Van Eetvelde, Jan Nyssen

**Affiliations:** Department of Geography, Ghent University, Ghent, Belgium; Department of Geography, University of Montenegro, Nikšić, Montenegro

**Keywords:** Montenegro, Land use changes, Marginal farmlands, Oral history, Qualitative research, Urbanization

## Abstract

Land use changes have been investigated in the surroundings of 14 rural Montenegrin settlements in order to get specific information about trends in land abandonment since around 1950. Permanently, seasonally and less inhabited settlements with different geographic conditions were studied. This was done by interviewing local inhabitants, which enabled a holistic approach to reveal the underlying processes of land abandonment. According to the observed patterns of land use change, the study sites can be categorized into intensified, urbanized, extensified, overgrown and forested cases. The category of extensified settlements is characterized by a highly reduced agricultural management intensity, resulting in an increase in grasslands and fruit trees at the expense of cropland. This land use change is mainly related to emigrating and aging inhabitants, having less livestock. Such extensive land use is found in both permanently inhabited and abandoned villages. Only some studied settlements became largely overgrown by bushes and forest. The steep average slope gradients and a large distance to the nearest city are explanatory factors of such land abandonment. Land use intensification takes place in low-lying areas located nearby towns.

## Background

A dominant trend of abandonment, reforestation and extensification has been found in the mountainous European areas since 1950, in line with predominant socio-economic changes through the continent (Mottet et al. 2006; Jepsen et al. 2015). The combination of environmental, economic and social aspects act as a driver for migration in rural contexts and it influences the land use changes (Kranjc 2008). More specifically, industrialization and urbanization have altered the landscape patterns since the Second World War, due to shifts in lifestyles, resulting in extensification of remote and physically disadvantaged rural areas and intensification and agricultural modernization of more urban areas (Antrop 2005; Klijn and Vos 2000). Especially rural, mountainous landscapes are determined as vulnerable to land abandonment (Baldock et al. 1996). Decrease or abandonment of ancient farming systems has caused an overgrowth of traditional farmlands by shrubs and trees (MacDonald et al. 2000). Such patterns have been registered in the mountainous regions of Southern Slovenia, where the traditional agricultural landscape was overgrown by forest by 1980. Relations of this trend to socio-economic factors have already been revealed: the emigration of rural inhabitants during the twentieth century and the accessibility of suitable lands to dwellings was detected as a driver of local agricultural intensification. Afterwards, some villages near access roads increased in terms of inhabitants but—as their activities are not related to agriculture anymore—this did not result in recent land use changes (Pausic and Carni 2012).

Traditional landscapes have been evolving gradually over time and were characterized by appropriate land use according to the local physical circumstances (Antrop 2000; Renes 2015). Initial settlements have often been established, taking into account land qualities and natural resources (LaGro 2001) as these provide inhabitants with ecosystem services on which they could rely for survival and welfare. According to Fagerholm et al. (2012), ecosystem services can be both materialistic (proper conditions for various crops or livestock, firewood, construction wood, wild fruits, herbs, etc.) as non-materialistic (places for enjoying nature, recreation, etc.). Inhabitants often possessed patches of land at different locations to have various physical circumstances (Antrop 2000) and a system of transhumance, where people seasonally migrated with their livestock in order to use grasslands in the mountains (Dodgshon and Olsson 2007). The human impact on the landscape visually occurs as lynchets, created by soil accumulated at the edges of parcels due to the long-lasting ploughing of croplands on slopes (Chartin et al. 2011), and forest cutting and picking of stones caused land to become sensitive to soil erosion, especially on hill slopes. When such human impacts diminish, the erosion degree reduces as forested, bushy and even grassy areas fix the soil and land resilience might take place (Nyssen et al. 2014). However, karst zones prove to be vulnerable to land degradation because they recover badly from degradation (Calo and Parise 2006).

Little scientific research has been performed concerning land abandonment factors within Montenegro. As this mountainous region is characterized by a variety of physical environmental settings and recently went through some turbulent socio-economic developments, it is able to indicate an interesting pattern of recent changes in land use and cover. Local-scale sites comprising different physical and demographic characteristics have been studied to obtain specific information about land abandonment. A holistic approach has been adopted as nowadays, many study methodologies are based on the integration of biophysical, environmental factors and socio-economic evolution to increase the understanding of landscape dynamics (Mottet et al. 2006).

## Methods

### Study area

#### Physical environment

**Fig. 1:**
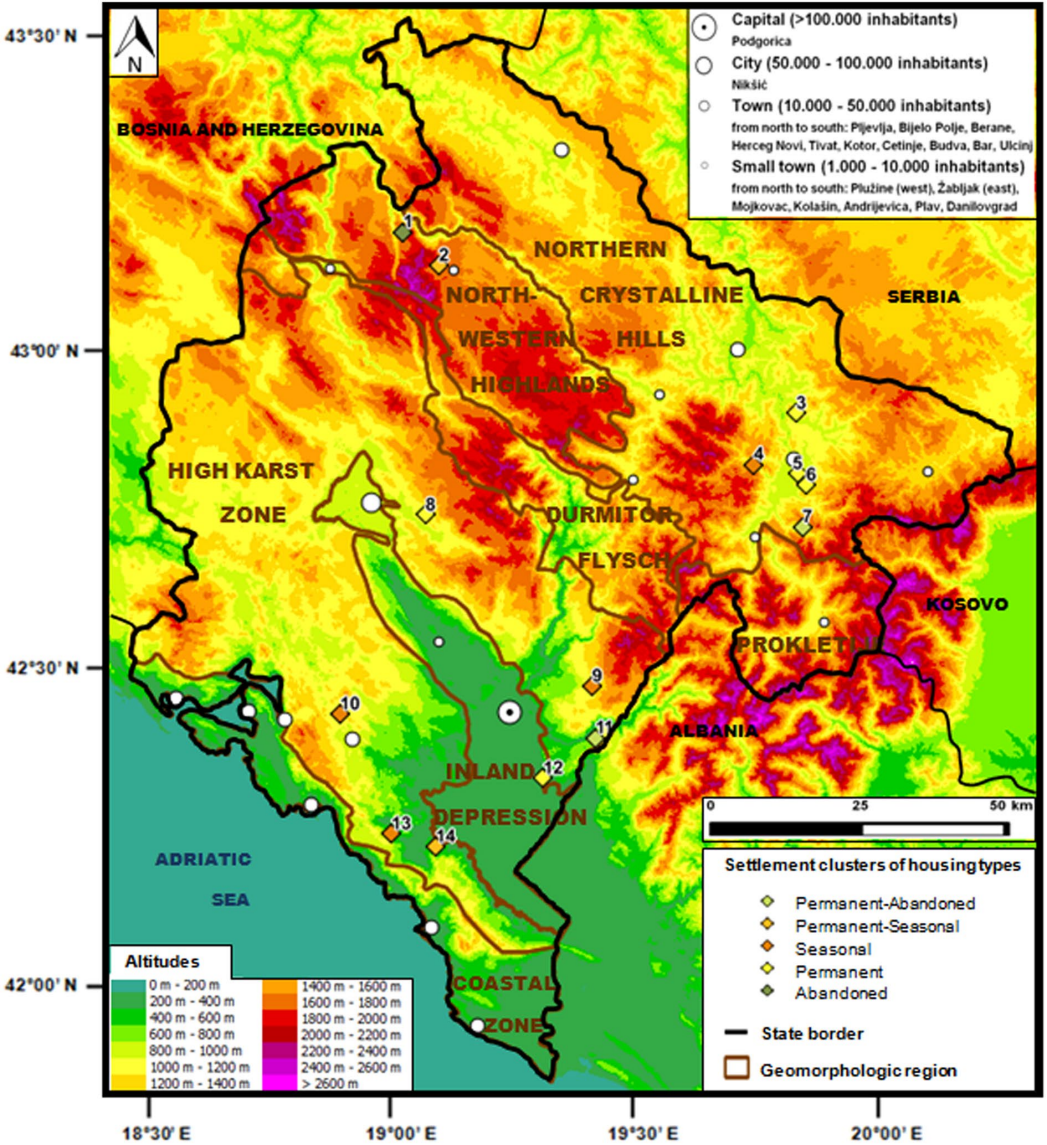
Selected settlements by cluster of housing type situated within distinct geomorphologic regions and with a range of altitudes and distances to the nearest town. *Source* geomorphic regions by Frankl et al. (2016)

Montenegro is situated in Southeast Europe along the Adriatic Sea and within the Dinaric Alps, a Western Balkan mountain range comprising mainly NW–SE oriented ridges (Kranjc 2008). The small (13,812 km^2^) country can be divided in seven geomorphological regions (Frankl et al. 2016; Fig. 1). The narrow *coastal zone* mainly contains small beaches with steep limestone slopes rising to average heights of 800 m, a ria coast centered on Boka Kotorska and a large debris cone near Albania on which sand beaches developed (Nyssen et al. 2014). NE of this zone lies the *high karst zone*: a dry Cretaceous limestone plateau characterized by karst phenomena, where the only fertile lands occur in poljes. This region is incised by the *inland depression,* which extends about 60 km in NW–SE direction (Mugoša 2008). It is mainly filled with Quaternary materials and comprises the polje of Nikšić, the area around the meandering river Zeta, the capital Podgorica, the lowland of Zeta Plain and Skadar Lake. Parallel with this zone the elevated *Durmitor Flysch* region is situated (often higher than 2000 m) with a relatively soft lithology dominated by sandstones, siltstones, marls and conglomerates. Furthermore, the *Prokletije* contains a varied geology (of schists, sand- and limestone, dolomites, volcanic outcrops, …) with glacial geomorphic features. The highest parts of this mountainous region extend to Kosovo and especially Albania. Within Montenegro, the *Northwestern Highlands* contain most peaks (with Bobotov Kuk being the highest: 2523 m a.s.l.) and plateaus. This region mainly contains limestone, its geomorphology is determined by a combination of glacial and karst processes (Annys et al. 2014). In the north, the Tara canyon marks the border with the *Northern crystalline hills*, which covers a large part of the country along the NE border and it mainly comprises flysch and sandstone sediments: softer materials forming an undulating landscape.

The climate of the coastal and high karst zone is Mediterranean with hot, dry summers and mild, wet winters (Nyssen et al. 2014) but precipitation is also affected by the mountain massifs (Kranjc 2008). The limestone hinterland is covered with typical bushy Mediterranean vegetation as maquis, garrigue and degraded evergreen woodland (Foster-Turley et al. 2010). The inland depression contains large rectangular vineyards (Zeta Plain) and some swampy areas (around Skadar Lake) and can be extremely hot during summer. The northern regions have a continental climate with rainfall, which is more equally distributed over the year, cold winters and warm summers. Forests and pastures cover large parts of these regions (Nyssen et al. 2014).

#### History and demography

Montenegro is characterized by a turbulent history. After hard times during the Second World War, the Socialist Federal Republic of Yugoslavia (SFRY) was reorganized in six republics: Bosnia and Herzegovina, Croatia, Macedonia, Montenegro, Serbia and Slovenia (Pittaway 2004). Industrialization occurred, causing migration to towns, urbanization and abandonment of mountain farming practices (MacDonald et al. 2000). After 1953, the short-lived Yugoslav agricultural collectivization faded away and gradually, farmers shifted from subsistence- to market-based production during the 1960s (Pittaway 2004). Then, industries of aluminum, steel and energy also became highly important as the transport infrastructure was growing (Miller 2005). In the early 1990s, the economic reformation towards an open market failed and as a result, a large decline in the Yugoslav economy took place (Lazic and Sekelj 1997). Then, the SFRY disintegrated (but Serbia and Montenegro temporarily stayed the last two members in the Federal Republic of Yugoslavia), defining the 1990s as the decade of Yugoslav wars (Miller 2005). Although Montenegro was located outside of the war zone, its general development suffered a lot. In 2006, the country became independent after a referendum that was narrowly won due to the Albanian minority (Pond 2006), but Montenegro (especially the north(east)ern part) remained strongly linked to Serbia (MONSTAT 2011a, b).

A strong population growth marked the twentieth century in Montenegro: from 311,000 inhabitants in 1921 to 620,000 at the beginning of the twenty-first century (MONSTAT 2011a, b). However, many internal migrations took place since around 1950, with a continuous emigration trend from the northern regions. The ratio of 46 % of the urban Montenegrins living in the northern regions in 1961 declined to about 30 % in 2000 (Mugoša 2008). Urbanization occurred in all 21 municipalities (due to migration, to their central settlement and mainly to Podgorica) (MONSTAT 2011a, b). Montenegro has 1256 settlements, 40 of which are urban. The coastal municipalities have the densest network of settlements, which have been analyzed for the presence of cultural and historical elements and forms of agricultural patterns (Curovic and Popovic 2014); especially the central area is economically important and highly urbanized, as the two largest cities (Podgorica and Nikšić, containing about one third of the population) are located there (MONSTAT 2011a, b).

#### Land use changes

Around 1900, Montenegro mainly consisted of bare land (Kranjc 2008), as only about 35 % of its landscape was densely vegetated. The coastal and central zones were especially barren (for about half of their area), while not even one tenth of the northern zone was barren wasteland, as it had more forests, meadows (respectively almost 30 and 40 %) and farmland (as opposed to small poljes and dry fields in the other areas). During the twentieth century, especially between 1950 and 1980, the Montenegrin landscape showed an overall increase in vegetation. The share of barren lands in the coastal and central zones strongly decreased in favor of shrubland and forests, while there was less vegetation growth in the northern zone, where forests grew at the expense of farmland, which halved at least (Nyssen et al. 2014). This evolution runs parallel with large sectorial shifts in employment, as the Montenegrins working in agriculture (about 75 % in 1948) fell back to 5 % in 1981 (Radović 1981). Restitution of lands to owners has also been playing a role in the decline of agriculture since the end of the 1980s (Mugoša 2008). However, due to the economic crisis in the 1990s, some marginal lands have been temporarily cultivated again (Grimes et al. 2005). Since 2000, areas occupied by infrastructure—often related to tourism—have rapidly increased in all Montenegrin regions (Nyssen et al. 2014) and regrowth of vegetation has accelerated. In 2008, dense forests covered 45 % of the country and another 9 % was under other woody vegetation types (including bushes, brushwood, maquis and stone steppes). By 2012, this had increased to respectively about 60 and 10 %, making Montenegro one of the most forested countries in Europe (Andelić et al. 2012).

### Selection of local-scale study sites

A variety of settlements in terms of demographic and physical characteristics has been selected to investigate land abandonment in relation to human lifestyles and their environment. Therefore, data about housing types per settlement (MONSTAT 2011a, b) were used; all dwellings have been categorized as permanently/seasonally occupied or abandoned. The share of housing types within a settlement defines it as permanent, permanent-seasonal, seasonal, abandoned or permanent-abandoned. A cluster analysis was carried out in SPSS so as to categorize 941 of all 1305 Montenegrin settlements in these five distinctive clusters. Settlements with more than 1000 houses, less than 10 houses and more than 25 % undetermined utilization types were excluded from the analysis. Besides representing a variety of settlement types, the selected settlements should be located in several of the geomorphological regions described by Frankl et al. (2016). Eventually, 14 settlements were chosen as study sites (Fig. 1; Table 1) representing all settlement types and four geomorphological regions (mainly the high karst zone and northern crystalline hills and—to a lesser extent—the northwestern highlands and inland depression).

**Table 1:** Overview of the locational and demographic features of the 14 selected settlements along with their cluster membership in the settlement typology

Settlement		Geographical position				Demographic characteristics										Settlement type/cluster
No.	Settlement name	Municipality	Coordinates (lat. °N; lon. °E)	Altitude (m)	Geomorphologic region	Number of permanent residents	Number of dwellings									
							Total	Permanent (%)		Seasonal (%)		Abandoned (%)		Other (%)		
1	Mala Crna Gora	Žabljak	43.207; 19.007	1450	NW highlands	53	84	28	(33)	10	(12)	44	(53	2	(2)	Abandoned
2	Bosača	Žabljak	43.161; 19.087	1550	NW highlands	20	18	11	(61)	5	(28)	2	(11)	0	(0)	Perm.-Seas.
3	Trubina	Bijelo Polje	42.935; 19.879	940	N crystalline hills	175	50	44	(88)	3	(6)	0	0	3	(6)	Permanent
4	Praćevac	Berane	42.850; 19.786	1100	N crystalline hills	31	50	14	(28)	33	(66)	0	0	3	(6)	Seasonal
5	Luge	Berane	42.836; 19.883	690	N crystalline hills	1841	683	515	(75)	68	(10)	5	(1)	95	(14)	Permanent
6	Zagorje	Berane	42.818; 19.904	840	N crystalline hills	243	101	72	(71)	22	(22)	6	(6)	1	(1)	Permanent
7	Orah	Berane	42.746; 19.896	1100	N crystalline hills	47	31	12	(39)	9	(29)	10	(32)	0	(0)	Perm.-Aban.
8	Oblatno	Nikšić	42.758; 19.072	860	High karst zone	96	28	23	(82)	4	(14)	0	0	1	(4)	Permanent
9	Orahovo	Podgorica	42.485; 19.442	900	High karst zone	81	723	44	(6)	671	(93)	1	0	7	(1)	Seasonal
10	Petrov Do	Cetinje	42.432; 18.892	870	High karst zone	9	38	5	(13)	23	(60)	9	(24)	1	(3)	Seasonal
11	Trabojin	Podgorica	42.399; 19.450	490	High karst zone	48	22	14	(64)	0	(0)	8	(36)	0	(0)	Perm.-Aban.
12	Vuksanlekići	Podgorica	42.336; 19.339	25	Inland depression	267	65	62	(95)	0	(0)	0	0	3	(5)	Permanent
13	Gornji Brčeli	Bar	42.240; 19.010	380	High karst zone	12	51	7	(14)	34	(67)	10	(19)	0	(0)	Seasonal
14	Godinje	Bar	42.221; 19.111	80	High karst zone	49	61	23	(38)	27	(44)	10	(16)	1	(2)	Perm.-Seas.

### Conducting and processing interviews

**Fig. 2:**
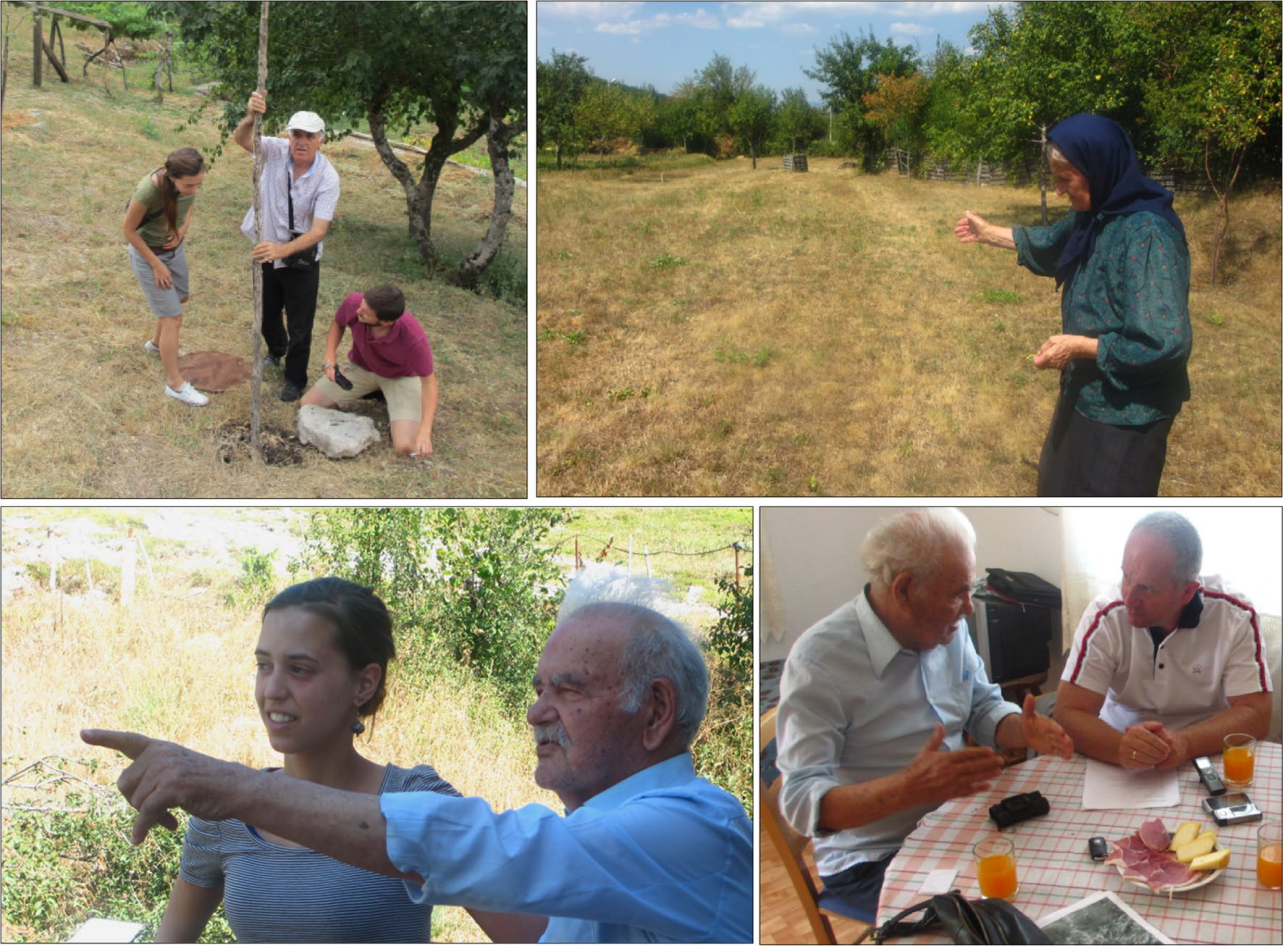
Participants as key informants of their own settlement area. **a** Interviewee showing a karst phenomenom on his property, Orahovo, 10/08/2013. **b** Interviewee showing her earlier cultivated area, Oblatno, 13/08/2013. **c** Interviewee showing the forest around his house, Petrov Do, 14/08/2013. **d** The typical informal setting for the conduction of interviews, Petrov Do, 14/08/2013

Semi-structured interviews have been conducted to gather first-hand information about land use/cover changes and thus land abandonment over time. The used questionnaire comprised six main topics: the basic characteristics of interviewees, agricultural situation and ecosystem services (both nowadays as in the past), aspects of land cover/use change (including matters as vegetation overgrowth and changes in the agricultural system, landscape and infrastructure), some physical geographical processes (like climate change and erosion), personal view and migration. Specific defined questions such as “How much livestock do you have now?” were not aimed at obtaining exact quantitative data but at facilitating answers and getting an idea about magnitudes. The questions ranged from completely open-ended to more narrow (although still open-ended). Participants were not forced to stick to the sequence of questions to prevent interruptions of their narratives. The used approach offers space to both theoretical ideas and empirical findings: the structure ensures the discussion of preconceived topics, while interviewees still have latitude to bring new aspects to the study (Galletta 2013). Interviewees were considered experts of their environment (Fagerholm et al. 2012) and the way of interviewing them provoked ‘oral histories’ (stories about specific memories related to the landscape), which helped to notice key periods of change, motivations, meanings and lifestyles (Galletta 2013). When possible, walks with informants were carried out to let them show interesting phenomena on the field (Fig. 2). Two to five interviews have been executed per settlement, until major study questions were answered (Neuman 2003). In total, 40 different (individual or group) interviews have been undertaken. As knowledge about landscape changes through recent history was required, mainly elderly people have been interviewed; approximately 70 % was older than 60 years.

All interviews were recorded, transcribed and loaded into the qualitative data analysis program NVivo to structure all data and to implement a constant comparison. This was done by using an open coding approach as included in the grounded theory procedures (Strauss and Corbin 1998). This means that relevant interview quotes were marked with codes of themes derived from literature as having a relation with land abandonment (Leech and Onwuegbuzie 2007). Afterwards, relations between coded quotes were explored by bringing them together per category and settlement in order to investigate the indicators of land abandonment.

### Quantification of the environmental characteristics

To examine the impact of the altitude, slope gradient, previous woody vegetation cover and distance to the nearest town, these environmental features were quantified. For the altitude, the elevation of the residential core of the village was taken. The slope gradient was measured by the creation of paths with height profiles on four transects (in N–S, NE–SW, E–W and NW–SE directions) through each study site (until the surrounding hill slopes) on recent satellite images of Google Earth. Subsequently, the sum of the absolute height differences in each transect was divided by its length to calculate the average slope gradient. Next, the woody vegetation cover was quantified using a point-counting method (Bellhouse 1981). This included the placement of a (10 × 10) regular grid (with 100 m between neighboring intersections) over Yugoslav topographic maps of the 1970s (on a scale of 1:25,000) of the settlement areas. For all intersections, it was recorded whether they corresponded with woody vegetation or not. Eventually, a percentage of woody vegetation cover was obtained for each study site. Lastly, the shortest distance to the nearest city (mostly the municipal capital) by road was measured in Google Earth.

### Rating the evolution of landscape elements and statistical analysis

From all collected data, quantitative values could be determined for the evolution of several landscape elements during the last few decades. These landscape elements have been classified into six categories: constructions (houses, barns, roads, etc.), cultivation (cropland, vineyards, gardens, etc.), grasslands (meadows and pastures), areas covered by fruit trees, bushy areas and forests. Measures for these parameters were scored on a bilateral scale, with −2.5 and 2.5 as respectively a large decrease and increase in the concerned factor and 0, meaning no change. This expert rating was done for each settlement using the interview transcripts, maps, satellite images and field observations. Afterwards, these values were used to create clusters with a similar change in land use/cover—settlement profiles—by cluster analysis. Finally, Pearson correlations between the quantified environmental factors and the measures of landscape changes (including trends of intensive and extensive land use and natural vegetation, comprising the summed scores of respectively the categories constructions and cultivation, grasslands, fruit trees and bushy area and forest), were calculated to explore their relations. Also, coefficients between these environmental factors were taken so as to check their mutual relations. Regression analyses have been carried out when conditions were met.

## Results

### Environmental characteristics and agricultural components

**Fig. 3:**
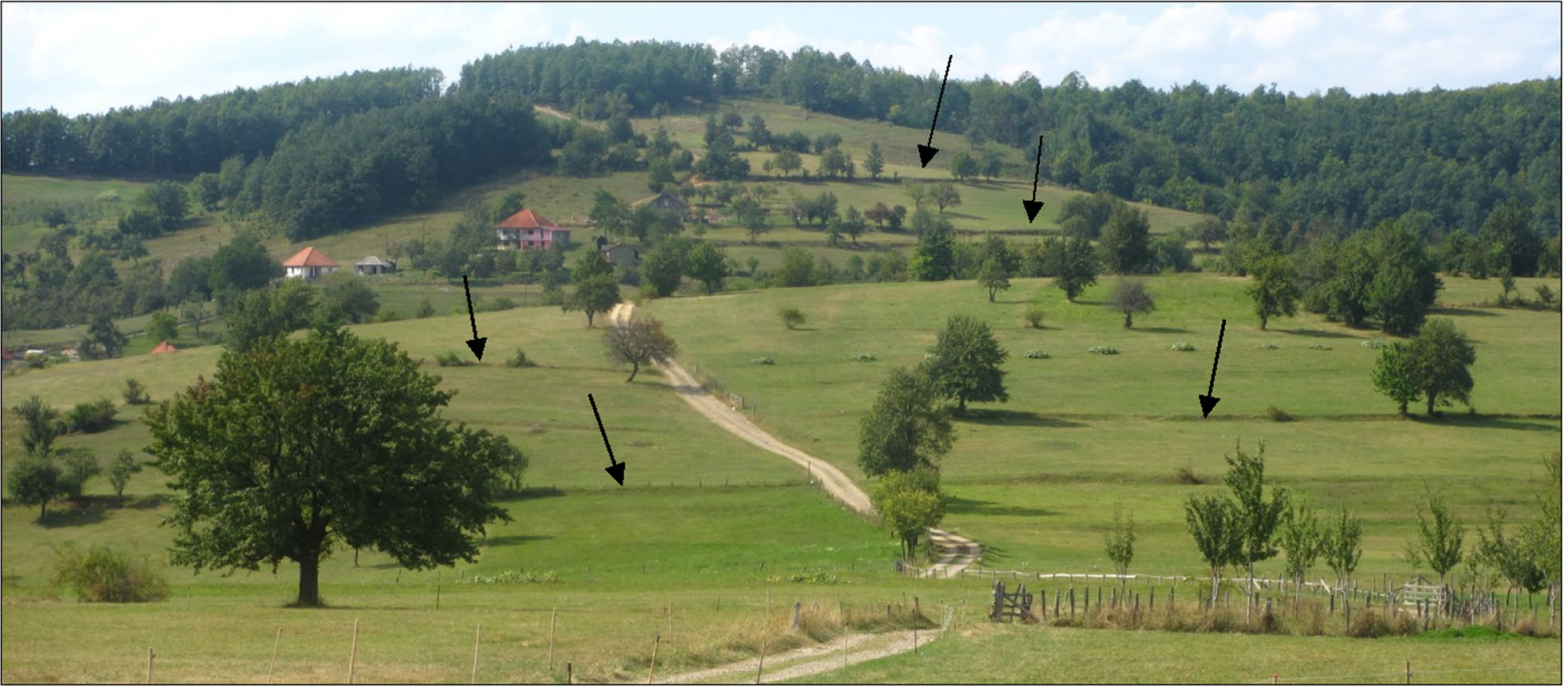
*Sklads* or lynchets on the grasslands of the settlement Trubina. Several *sklads* are marked by an *arrow*. 01/09/2013

The selected settlements show a variety in altitude, slope gradient, previous woody vegetation cover and distance to the nearest town (Table 2). Furthermore, some concepts typical for rural Montenegrin areas emerged from the interviews. Firstly, the *katun* was mentioned, a complex of mountain pastures with extensive common meadows (and often cottages), where inhabitants lived during summertime with their livestock. Some settlements had several *katuns*, located at different altitudes, which were used in sequence. Nowadays, many of them are not used anymore and became (partly) abandoned. Also, *lynchets*, nowadays mainly under grassland, prove the previous existence of croplands on slopes (Fig. 3). Cultivation in *dolines* was encountered in study sites on limestone but nowadays, these only subsist as small gardens. The production of *lime* and *charcoal* was traditionally performed in calcareous regions with a degraded forest but has almost been abandoned.

**Table 2:** Geographic parameters altitude, average slope gradient, woody vegetation cover in the 1970s and distance to nearest town for each settlement

No.	Settlement name	Altitude (m)	Average slope gradient (%)	Woody vegetation cover in the 1970s (%)	Distance to nearest town (km)
1	Mala Crna Gora	1450	10.0	5	18
2	Bosača	1550	13.5	56	5
3	Trubina	940	13.0	64	16
4	Praćevac	1100	27.5	56	12
5	Luge	690	8.0	25	2
6	Zagorje	840	16.5	45	7
7	Orah	1100	26.0	43	17
8	Oblatno	860	12.0	57	12
9	Orahovo	900	14.5	26	21
10	Petrov Do	870	8.5	27	14
11	Trabojin	490	21.5	62	25
12	Vuksanlekići	25	8.0	7	12
13	Gornji Brčeli	380	22.5	55	25
14	Godinje	80	22.0	43	25

### Trends in the settlement areas

While comparing all interview transcripts, several predominant aspects have been detected. About 80 % of the interviewees mentioned farming as a (former) secondary job and 10 % as their main employment. Most of them are still cultivating vegetables and fruits for personal use. The number of livestock has strongly decreased (for almost everyone) since 50 or even 2 years earlier; nowadays most inhabitants have none to three cows, some have about 10–30 sheep but almost no one still owns a horse or ox. About 70 % of the interviewees stated a large difference in territorial use: a more extensive instead of intensive land use with less cropland, more (maintained) grasslands and fruit trees or more overgrown bushy areas. Most interviewees go to town less than five times a month to visit shops, banks and doctors, as their settlements do not have these facilities. Almost all interviewees mentioned getting a (small) pension from the state or from the country where they had worked temporarily. More than 90 % of the children of the interviewees had migrated to an urban area; about half of them moved abroad and the other half to the Montenegrin cities. Almost all participants told they preferred living in their settlement over an urban lifestyle. Besides, many of them said that it is easier to farm and live rurally than before but less people (especially youngsters) want this. Furthermore, in all studied villages, inhabitants own lands with different kinds of land cover; (almost) each one has a garden around the house, meadows on different altitudes and one or more plots of forest. A diminished soil quality (due to a decrease in livestock) has also frequently been mentioned. Furthermore, many interviewees stated that the summers became hotter and drier than before. Also, the inflations of the 1990s caused no large changes or problems in rural settlements, as inhabitants were relatively self-sufficient. Specific features and trends for settlements are given in Table 3.

**Table 3:** Statements emerged from the interviews about several situations in the studied settlement areas, with + as confirmation, – as negation, / as not determined or relevant and the numbers 1–14 representing the settlements (see Tables 1, 2)

Statement	Settlement number													
	1	2	3	4	5	6	7	8	9	10	11	12	13	14
Land is mainly used for grass cutting instead of crops	/	+	+	+	+	+	+	+	+	/	+	−	−	−
Most fields are abandoned and overgrown by bushes	+	−	−	+	−	−	+	−	−	−	−	−	+	+
The *katuns* in the mountains are still used nowadays	+	/	+	−	+	+	−	−	−	/	−	/	−	−
Now there are more forested areas around the village	+	+	+	−	+	+	+	−	−	+	−	−	+	+
… because people cut less trees now	+	+	−	−	+	+	+	+	−	+	+	+	+	+
There is more forest but of less quality now than before	/	−	+	+	−	+	−	+	+	/	/	−	−	−
There is more degraded forest now due to forest fires	−	−	−	+	−	−	−	+	+	+	+	+	−	−
There are high quality trees for construction wood	+	+	−	+	+	+	+	−	−	−	−	−	−	−
Residents still collect mushrooms or berries in the forest	+	+	+	+	+	+	+	+	−	−	−	−	+	+
Residents made *kreč* or charcoal with degraded wood	−	−	−	−	+	−	−	+	+	+	+	+	+	+
Now there are more (fruit) trees in the village	/	/	+	+	+	+	+	+	+	/	+	−	+	+
There are erosion processes	−	−	−	−	+	−	+	+	+	+	+	+	+	−
There are karst phenomena	+	+	−	−	−	−	−	−	+	+	+	−	+	−
People cleaned farmlands of stones and made walls of it	+	−	−	−	−	−	−	+	+	+	+	−	+	+
There are terraces	−	−	−	−	−	−	−	−	+	+	+	−	+	+
There are lynchets	−	−	+	+	+	+	+	+	−	−	−	−	−	−
It is still difficult to survive due to the lack of water	+	−	+	−	−	−	−	−	−	+	+	−	−	−
Residents built (a part of) their roads or electricity	+	−	/	/	−	−	+	−	−	+	/	−	+	−
Residents still burn their garbage or throw it in the river	+	+	+	+	−	−	+	+	−	+	+	−	−	−
There is a pro-Serbian mentality in the settlement	−	−	−	+	+	+	+	+	+	/	−	−	−	−
The main emigrations were in the 1970s	−	+	−	+	/	+	+	+	+	+	+	+	+	+
The main emigrations were in the 1990s	+	−	+	−	/	−	+	+	−	−	−	+	−	−
Now only the elderly people remain to live here	+	−	−	+	−	−	+	+	+	+	+	−	+	+
Only a few families live here during the winter	+	−	−	+	−	+	−	−	−	+	−	−	+	+

### Land abandonment factors and processes

#### Location

The distance to urban areas influences the land use change. Large cities (as Podgorica) have a larger sphere of influence than smaller towns (as Žabljak). Urban–rural gradients of study sites include Luge, Zagorje (both permanent), Praćevac (seasonal) and Orah (permanent-abandoned) to the city Berane, Bosača (permanent-seasonal) and Mala Crna Gora (abandoned) to the town Žabljak and Vusanlekići (permanent) and Trabojin (permanent-abandoned) to Podgorica. Generally, the closer a village is situated to a town, the less abandoned it became. Within permanent(-seasonal) inhabited study sites (as Trubina and Bosača), abandoned and overgrown grasslands are generally situated at the edges of the village, because parts of the land had been exchanged between staying and migrating family members. However, when settlements became more abandoned (as Mala Crna Gora), overgrown areas appear on their territories everywhere.

#### Physical geography

**Fig. 4:**
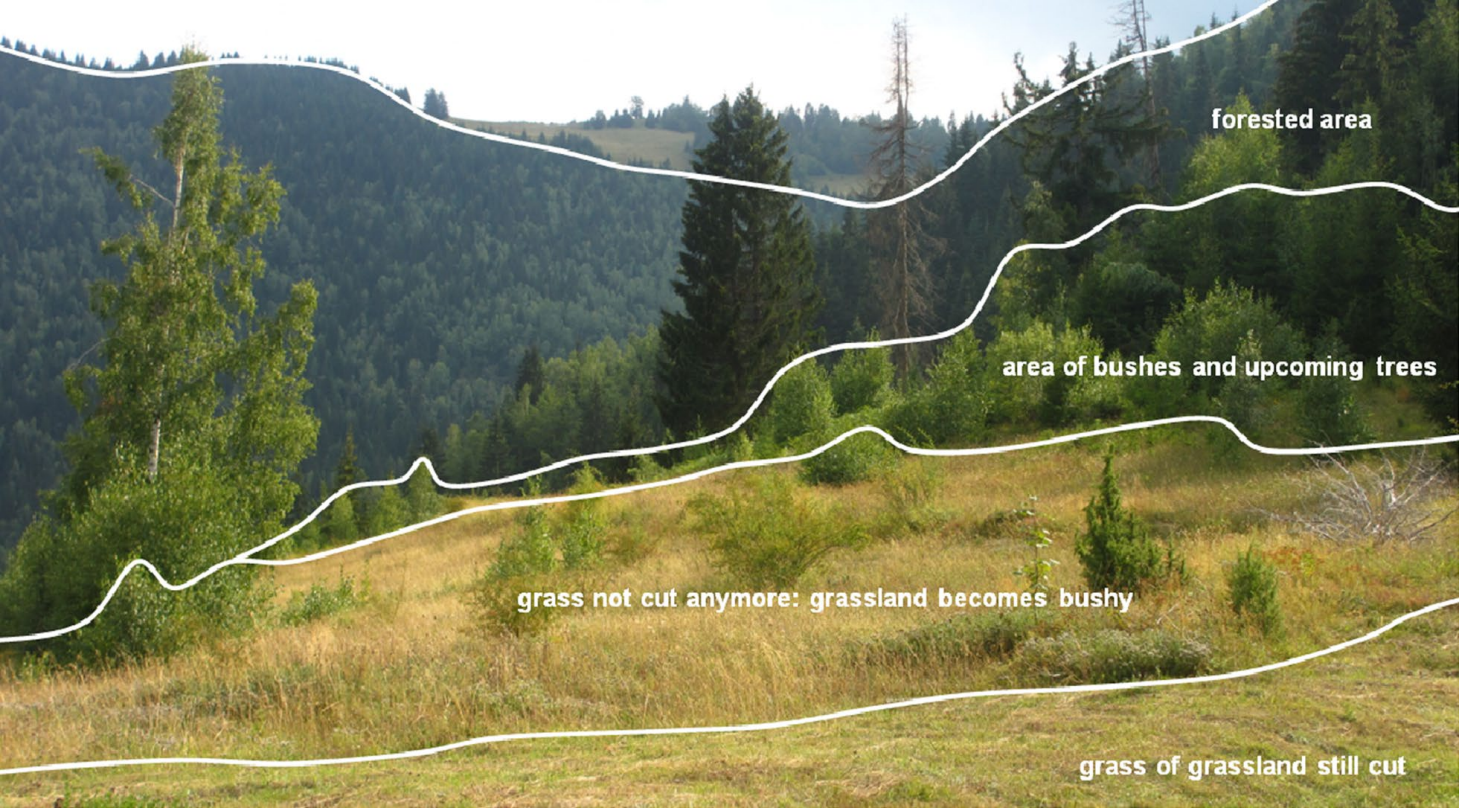
Phases in the process of overgrowth of grasslands to natural vegetation in the katun Ravni, as not all lands become abandoned at the same time, Praćevac, 02/09/2013

**Fig. 5:**
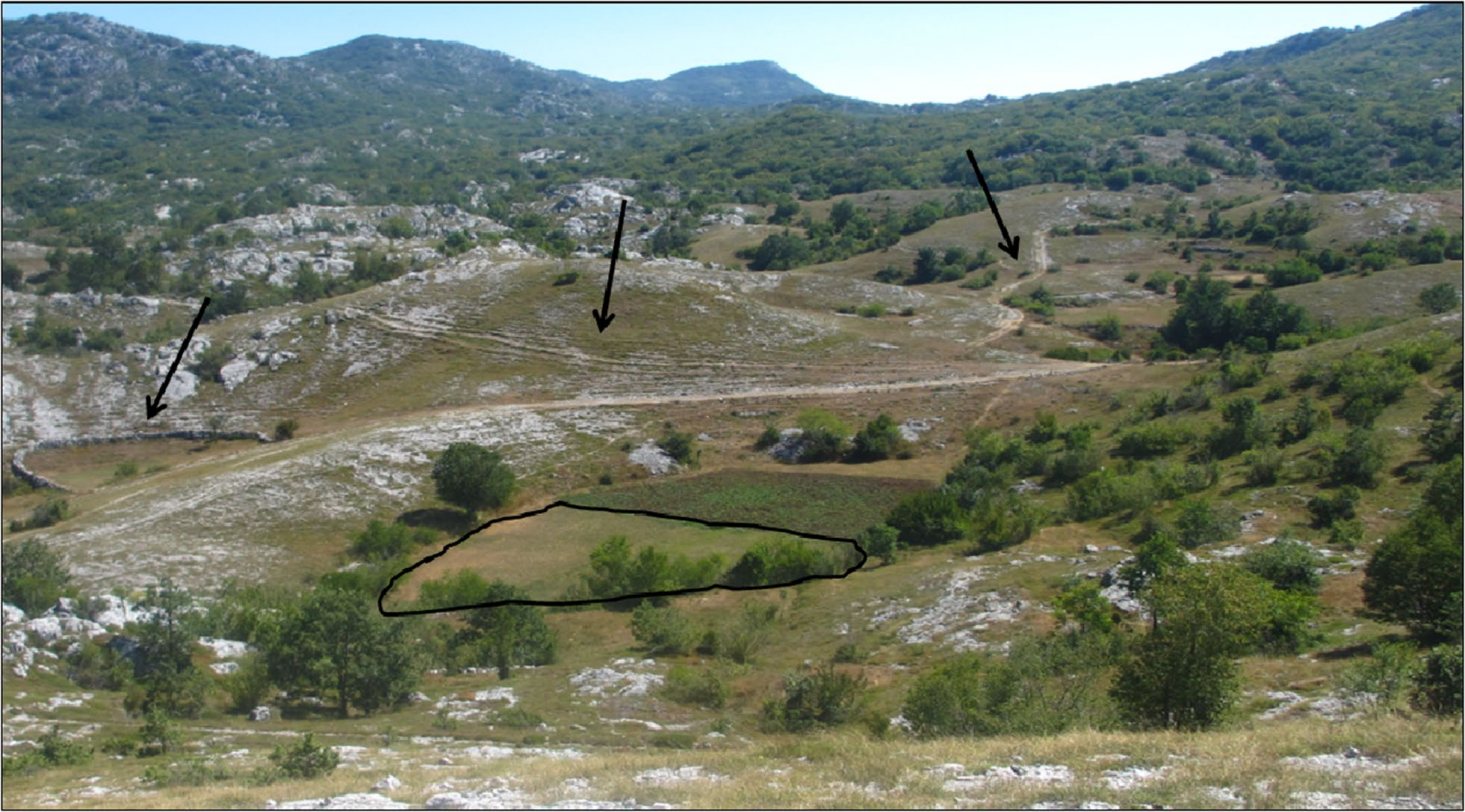
Half cultivated garden in a doline (*foreground*). Livestock is still kept in this landscape, as evidenced by the presence of tracks and a pen (*arrows*). Petrov Do, 14/08/2013

The altitude determines the presence of *katuns* and thereby land abandonment, as these elevated areas often were abandoned first. For example, the *katuns* of Praćevac are at the first level partially (Fig. 4) and at the second level, totally abandoned and overgrown. Furthermore, the often mentioned hotter, dryer and longer summers cause more dry soils nowadays. In parts of the Montenegrin karst region, water has always been the main restriction for agriculture as cultivable soil is very scarce and thus all small fertile grounds (dolines) were (and still are) well maintained (as in Petrov Do, Fig. 5). Other areas (as Godinje and Gornji Brčeli) have springs and more fertile soils, which became quickly overgrown due to bad maintenance and abandonment. Usually, the best grounds are being kept, while less fertile lands had been abandoned or exchanged. In Bosača, the latter was done with lands in Pitomine (a neighbouring village), which have a lower soil quality. Also, the slope gradient influences the land abandonment. In several villages, interviewees told that abandoned and overgrown lands are mainly situated on steep slopes, while these areas were traditionally meant for grasslands (rather than croplands).

#### Vegetation and ecosystem services

Several study sites have forests with high quality trees (mainly pines) which have value as timber and therefore, inhabitants do not ruin them by making charcoal or lime, which was done in regions with a degraded, bushy vegetation. Some interviewees own separate forest patches with trees for firewood (lower situated) and high quality trees (located higher). People always need permission and tax payment for logging. Generally, all settlements are more vegetated now; mainly the areas around the houses became greener because of an increase in fruit trees, grasslands and bushy areas at the expense of croplands. Therefore, most settlements gained a more closed landscape without attributing this to abandonment alone. In Zagorje, the central plain traditionally comprised open space but during the 1980s, new houses appeared with an increase in gardens and plum trees. The study sites Gornji Brčeli and Godinje show many new bushes and trees due to the abandonment of croplands on cultivation terraces, leading to an almost inaccessible vegetated area. A result of the emerging forest is the increased amount of mushrooms, berries and herbs in these settlements, enabling more inhabitants to collect and sell them.

#### Agriculture

Nowadays, physical circumstances of territories are less determinative than before, as agricultural tools and inputs have drastically improved and local inhabitants do not need much space for farming anymore. Some interviewees mentioned people respected their land more in the past by using the unfertile areas for building and keeping fertile grounds for cultivation. For example, the settlement structure of Zagorje evolved from houses only being located at the surrounding hill slopes to the scattered habitat over the territory; also after the devastating earthquake of 1979 in Godinje, a part of its traditional houses on the hill slopes have been rebuilt on the lower, flatter area used for vineyards earlier on. Furthermore, in the past, almost every house had one or two oxen for land cultivation. Herds of sheep, goats or cows were taken to meadows around the village or the *katuns*. Also, interviewees in Godinje mentioned the former occurrence of stubble grazing on the residues of the harvested croplands. Nowadays, mainly gardens around houses remain areas of intensive land use. Croplands for fodder are no longer necessary, as inhabitants do not have much livestock anymore. A transition from croplands to grasslands, bushy areas and eventually forests is generally observed. However, not all grasslands became overgrown, as several inhabitants were able to maintain them.

#### Demography

Demographic changes due to emigration and aging appeared to be the major driving forces for land use change. Most family sizes reduced as many people in their twenties migrated to the cities (mainly their municipal capital, Podgorica or a coastal city) or abroad (often Serbia and some to another ex-Yugoslav country), while the old, remaining family members were passing away. Usually, these remaining persons only cultivate according to their needs and thus, as a result, gardens became smaller. Their old age and the absence of helpers also determine the declining amounts in livestock and crops. One of the reasons for emigration nowadays is that young people cannot find a partner if they stay in their village, as many traditional settlements only house few different families. Furthermore, it was frequently heard that inhabitants temporarily migrated to other nearby countries (mainly Germany, Austria or Italy) to work some time for a higher salary. With less people, less land is cultivated or maintained, more areas become overgrown and less trees are cut for firewood in the forests surrounding the villages. Large variations occur between properties of seasonal inhabitants. Owners are often there during summers, weekends or vacations and they maintain their lands well, while others almost never come, have much overgrown land or let neighbours maintain it.

#### Society

Lastly, policy and laws have an impact on the land use change. The establishment of national parks largely influenced the forest policy in the concerned areas. For example, due to the more strict laws of the Durmitor National Park since the 1980s, the surroundings of Mala Crna Gora became more forested. In several areas which are unprotected as a national park, the laws on tree cutting also became stricter according to interviewees. Since the last 20 years, the authorities have been acting stricter and did not allow firewood cutting for sale; permissions to cut trees in private forests are harder to obtain and there is much forest owned by the state. However, interviewees of some settlements (like Orahovo, near Podgorica) mentioned that nowadays, authorities are tolerating the illegal cutting of good quality forest (facilitated by better machinery, including second-hand military vehicles), which did not happen until 1990. Furthermore, war periods and economic crises had an impact on the environment. The inflation during the 1990s, coupled with weakness of authority, caused more mushroom picking in forests and tree cutting as people needed to be more self-sufficient. Then, there were -about one third- less trees in Bosača, due to the uncontrolled cutting. The collective agricultural system of the early 1950s, when people had to give away their lands, is partly responsible for the many overgrown terrains in Godinje, as these unclaimed lands remain state property until the hypothetical previous owners are claiming them back.

### Settlement profiles of landscape changes

**Fig. 6:**
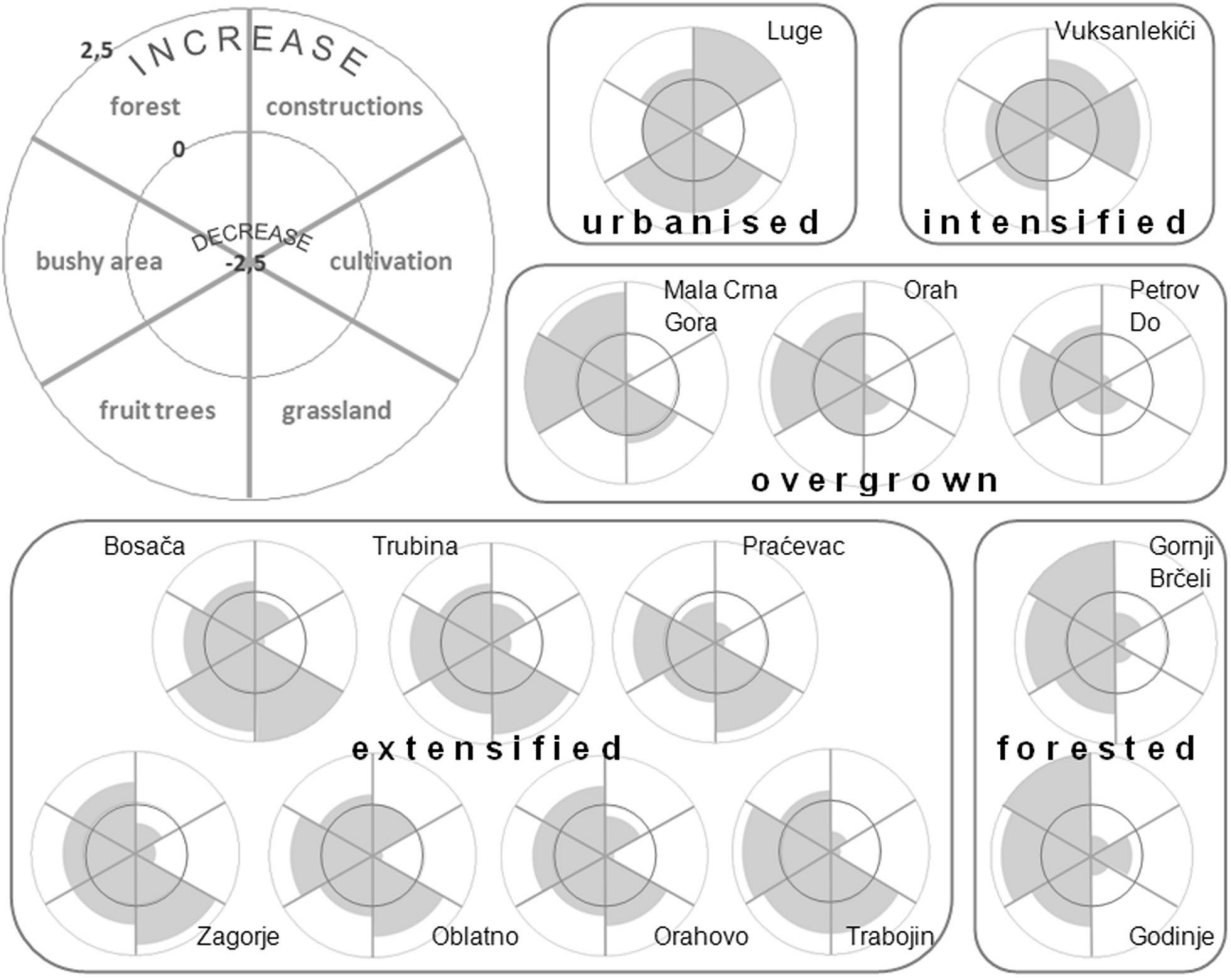
Pie radar charts showing settlement profiles of land use changes

The evolution in land use change of all 14 study sites can be clustered into five distinctive groups of land use change (Fig. 6; Table 4). The first two settlement profile types—urbanized and intensified—both contain only one settlement—respectively Luge and Vuksanlekići. Luge is characterized by an obvious increase in construction but not in cultivation, while Vuksanlekići largely increased in cultivation and -to a much smaller extent- in construction. All other settlements are characterized by a decrease in intensive land use, while most of them show an increase in extensive land use. Seven settlements—Bosača, Trubina, Praćevac, Zagorje, Oblatno, Orahovo and Trabojin—are categorized as extensified and characterized by a significant decrease in intensive land use (mainly in terms of croplands) on the one hand and a strong increase in extensive land use (as grasslands and fruit trees) on the other hand. Moreover, they contain recently grown shrubs, bushes and trees. The overgrown settlement profile includes Mala Crna Gora, Orah and Petrov Do and their territories, where intensive land use has strongly decreased but also (slightly) extensive land use (fruit trees and mainly meadows). Furthermore, some surfaces have recently become overgrown by natural vegetation. Finally, Gornji Brčeli and Godinje were heavily forested during the last decades. These settlements have a decreased area of intensively used land, as well as grasslands.

**Table 4:** Settlement profiles with a short description of the most important trends

Cluster name	Settlements	Description
Urbanised	Luge	Much more houses, more small gardens and fruit trees, much less cropland
Intensified	Vuksanlekići	Much more vineyards, some more houses, much less grassland
Extensified	Bosača—Trubina—Praćevac—Zagorje—Oblatno—Orahovo—Trabojin	Little less number of houses, often with smaller gardens, much less cropland, more meadows and fruit trees
Overgrown	Mala Crna Gora—Orah—Petrov Do	Much less houses and cultivation, more bushy areas, some more forest
Forested	Gornji Brčeli—Godinje	Less houses and cultivation, less grass land, much more bushy area and especially recent forest

### Comparison of the variables

According to the calculated Pearson correlation coefficients, the average slope gradient is moderately related to the previous woody vegetation cover (r = + 0.57) and weakly to the distance to the nearest town (r = + 0.47). The factor ‘altitude’ is weakly related to the distance to town (r = −0.38). Furthermore, correlations between the geographic features and land use changes are investigated, where the former aspects are expected to explain some values of the latter (Table 5). The strongest correlations are detected between the altitude and changes in the cultivated area (r = −0.73), slope gradient and changes in the bushy area (r = + 0.71) and distance to the nearest town and changes in the bushy area (r = + 0.69). The change in the constructed area is significantly correlated with the distance to town (r = −0.55), as well as the slope gradient (r = −0.50). The changes in forest and extensive land use appear to be rather unrelated to the studied geographical aspects, with only a moderate correlation (r = + 0.54) between the previous vegetation cover and the change into grassland.

**Table 5:** Matrix comprising Pearson correlation coefficients of possible determining geographical variables (rows) and influenced variables of land use changes (columns)

Independent variables: geo-graphic aspects	Dependent variables: land use changes								
	Construction	Cultvation	Intensive land use	Grassland	Fruit trees	Extensive land use	Bushy area	Forest	Natural vegetation
Altitude (m)	−0.28	–0.73***	–0.60**	0.40	–0.19	0.24	0.18	–0.08	0.04
Slope gradient (%)	−0.50*	−*0.26*	−0.47*	*0.11*	0.25	0.17	0.71***	0.42	0.62**
Vegetation (%)	−0.27	−0.35	−0.37	0.54**	0.37	0.55**	*0.23*	0.13	0.20
Distance (km)	−0.55**	*0.04*	−*0.32*	−0.36	−0.28	−0.38	0.69***	0.34	0.56**

A underline font shows that conditions for linear regression analysis are satisfied, while an italic font shows that this is not the case. The asterisks show the significances of the executed linear regression analyses, with * *p* < 0.10; ** *p* < 0.05, *** *p* < 0.01

Lastly, when combining all settlement clusters and profiles, a large variation in housing type (settlement clusters) exists for the extensified settlement profiles, having mainly permanent and seasonal settlements. The overgrown settlements are mainly (partly) abandoned and the two forested settlements are seasonal and permanent-seasonal.

## Discussion

### Land use changes

The studied areas show similar trends, as well as variations concerning the land use change. All rural inhabitants still own differently located territories, but while in the past the purpose was to enable several types of land use (Antrop 2000), now less strong relations between land use and soil qualities exist due to the declining cultivation activities. However, cultivating in dolines and maintaining them in the limestone areas (i.e. using their ecosystem services) is still occurring when there is a need for it. In Montenegro, firewood remains the dominant type of fuel for heating, especially in (northern) rural settlements and about 14 % of the households get firewood from their own forest (Glavonjic and Krajnc 2013). Furthermore, lynchets situated in the meadows and terraces overgrown by bushes indicate the decrease of former agricultural practices and the shift in land cover. The land use changes were found to be highly related to the shifts in lifestyles as mentioned by Fry (2000) with social, economic and political driving forces. However, ambiguities exist about the role of the authorities in logging. Some interviewees mentioned more logging in the forests around their village in the early 2000s than before, due to the better machinery and a lack of control. Conversely, in other settlements, interviewees talked about the current stricter laws to cut trees in their own forests and thus the (forced) decrease of it. A possible explanation for this are the stricter laws in the private forests—which significantly increased during the last decades to about 33 % in 2008 (Andelić et al. 2012)—against the inefficient forest management of the state forests (Grimes et al. 2005). However, the forest management became more efficient in controlling the illegally obtained timber wood through clear cuts and wildfires since 2000 according to Foster-Turley et al. (2010). Furthermore, the conclusion of Nyssen et al. (2014) about the general increase of the forest in Montenegro throughout the twentieth century can be more nuanced according to this research. In some sites, it was said that—although there is more vegetation now—the quality of the forest has decreased. Also, many interviewees mentioned that—mainly inside their settlement—the vegetation increased at locations where they cultivated the land before, creating a more closed local landscape. Research in other topographically complex areas (such as the Alps) found that the cultivated land in particular is affected by large socio-economic changes (Schirpke et al. 2012).

### Land abandonment versus extensification

The most frequently observed phenomenon was the decrease of (intensively) cultivated areas and the increase of (extensively) maintained grasslands and fruit trees. Since most inhabitants have less livestock now, they do not need crops for fodder anymore to feed them efficiently. Therefore, a less intensive method is achieved by giving hay through grass cutting on former croplands. As two cows need lots of hay, large meadows have to be maintained. Also, several inhabitants maintain their grasslands only in order to keep their property tidy. Because of the aging of the remaining rural inhabitants, it is not evident for them to do intensive labour anymore. Therefore, fruit trees became popular too, as this represents also a more extensive way of gardening. These findings are comparable with the dominant trend of extensification in the mountainous rural European areas since about 1950 (Mottet et al. 2006). The single significant relation between the increasing grasslands and the woody vegetation cover in the 1970s can be partially explained through the good soil characteristics for vegetation and the intermediate effect of the slope gradient. Land abandonment in terms of an enormous increase in natural, wild vegetation was found to a lesser extent, although in all extensified villages several parcels became so. The overgrown territories turned out to be mainly influenced by their location (far from a city), slope gradient (steep) and altitude (elevated) and have the most abandoned houses. The forested territories are influenced by the same geographical factors but are situated not so high and house more inhabitants that are seasonal.

### Interview methods

Interviewing local inhabitants provided us with useful, detailed data about their lifestyles and habits in particular. However, sometimes contradictory comments were given, so interviews had to be analyzed critically. Indications for reliability were the recurring similar answers by several interviewees of the same settlement. It was easier to ask interviewees detailed questions about their personal experiences than about general landscape evolutions. Also, it was more interesting to interview men, since married women had migrated to the village of their husband and they did not know the area since childhood as a result.

## Conclusion

The evolution of land abandonment in rural Montenegro since about 1950 at 14 local study sites and their underlying processes has been examined. Certain trends can be partially explained through geographical conditions. Settlements with steep slope gradients and large distances to cities show an increase in overgrown, bushy lands, while the cultivated area decreased for villages at high altitudes, and small distances to cities led to more house construction. Also, demographic and social circumstances demonstrated their impact on the landscape. Villages where lands were extensively maintained turned out to be mainly characterized by emigration and aging of their inhabitants; such extensification was also significantly correlated with the woody vegetation cover in the 1970s. Five settlement profiles could be distinguished: urbanized, intensified (both comprising only one settlement), extensified, overgrown and forested types of settlements. Most study sites displayed a trend of extensification, with maintained grasslands and fruit trees instead of traditional croplands and much livestock. Also, often areas became overgrown by bushes and vegetation, mainly at edges and on mountain areas of settlement territories, but also centrally within villages overgrown grasslands occur. These processes -along with the increase of fruit trees- caused a shift from rather open to more closed landscapes within settlements.

Public attitudes towards such “rewilding” processes (sensu Bauer et al. 2009) and social-ecological benefits (Navarro and Pereira 2012) should be studied in order to develop policies to cope with the phenomenon of overgrown village territories that occurs in many mountainous regions in Mediterranean Europe.
